# HnRNP L is important for the expression of oncogene SRSF3 and oncogenic potential of oral squamous cell carcinoma cells

**DOI:** 10.1038/srep35976

**Published:** 2016-11-03

**Authors:** Rong Jia, Si Zhang, Miaomiao Liu, Yan Zhang, Yu Liu, Mingwen Fan, Jihua Guo

**Affiliations:** 1 Hubei-MOST KLOS & KLOBME, School & Hospital of Stomatology, Wuhan University, Wuhan, PR China; 2 College of Life Sciences, Wuhan University, Wuhan, PR China

## Abstract

Oral squamous cell carcinoma (OSCC) is the leading cause of death related to oral diseases. The mechanisms of OSCC development remain largely unknown. Heterogeneous nuclear ribonucleoprotein L (HnRNP L) is a multi-functional splicing factor. It has been reported to be an important regulator of apoptosis. However, the functions of hnRNP L in cancer need to be further explored. In the present study, we found that OSCC tissues expressed significantly higher levels of hnRNP L than normal tissues. Depletion of hnRNP L retarded cell growth, cell migration, and tumorigenesis of OSCC cells. HnRNP L regulates both the expression of oncogenic splicing factor SRSF3 and the alternative splicing of SRSF3 exon 4. Expression of hnRNP L is correlated with SRSF3 expression in OSCC tissues. These findings suggest that hnRNP L is important for the pathogenesis of OSCC and may be a novel potential therapeutic target of OSCC.

Oral squamous cell carcinoma (OSCC) is one of the most frequent human malignancies, which accounts for 90% of all oral cancers^1^. The mortality rate of OSCC is still high despite recent advances in treatment protocols, including chemotherapy, and radiotherapy^2^. In addition, the underlying molecular mechanisms of OSCC development remain largely unknown. Thus, there is an urgent need to identify novel therapeutic targets for OSCC.

Most mRNA precursors of human genes undergo alternative splicing. Misregulated alternative splicing of pre-mRNA is increasingly linked to tumorigenesis^3^. Splicing factors play key roles in regulating the alternative splicing of pre-mRNA. Several splicing factors have been shown to be oncogenic, including SF2/ASF^4^, SRp20^5^, and SRSF6^6^. Accumulated evidences suggest that aberrant expression of splicing factor is associated with cancers^7^,^8^.

HnRNP L is a multifunctional splicing factor. It participates in a series of RNA-related processes, including chromatin modification^9^, export of intronless mRNAs^10^, regulation of alternative pre-mRNA splicing and poly(A) site selection^11^,^12^, translational regulation^13^,^14^, and mRNA stability^15^. Knockout of hnRNP L leads to altered hematopoiesis and premature death^16^.

HnRNP L has been reported to be involved in tumorigenesis. Goehe *et al*.^17^ found that down-regulation of hnRNP L significantly affected tumorigenic capacity in a non-small cell lung cancer cell line *via* apoptosis. A proteomics study showed that the expression level of hnRNP L in esophageal cancer cell line is over five-fold higher than that in an immortal cell line^18^. However, the expression and functions of hnRNP L in tumors remain largely unknown.

In the present study, we found that hnRNP L is significantly overexpressed in OSCC tissues compared with normal oral mucosal tissues. HnRNP L is important for OSCC cell growth, cell migration, and tumorigenesis. Oncogenic splicing factor SRSF3 is a novel target of hnRNP L. Our results uncovered new characteristics of hnRNP L in tumorigenesis and its essential target.

## Results

### HnRNP L is overexpressed in OSCC tissues and cells

**Figure 1:**
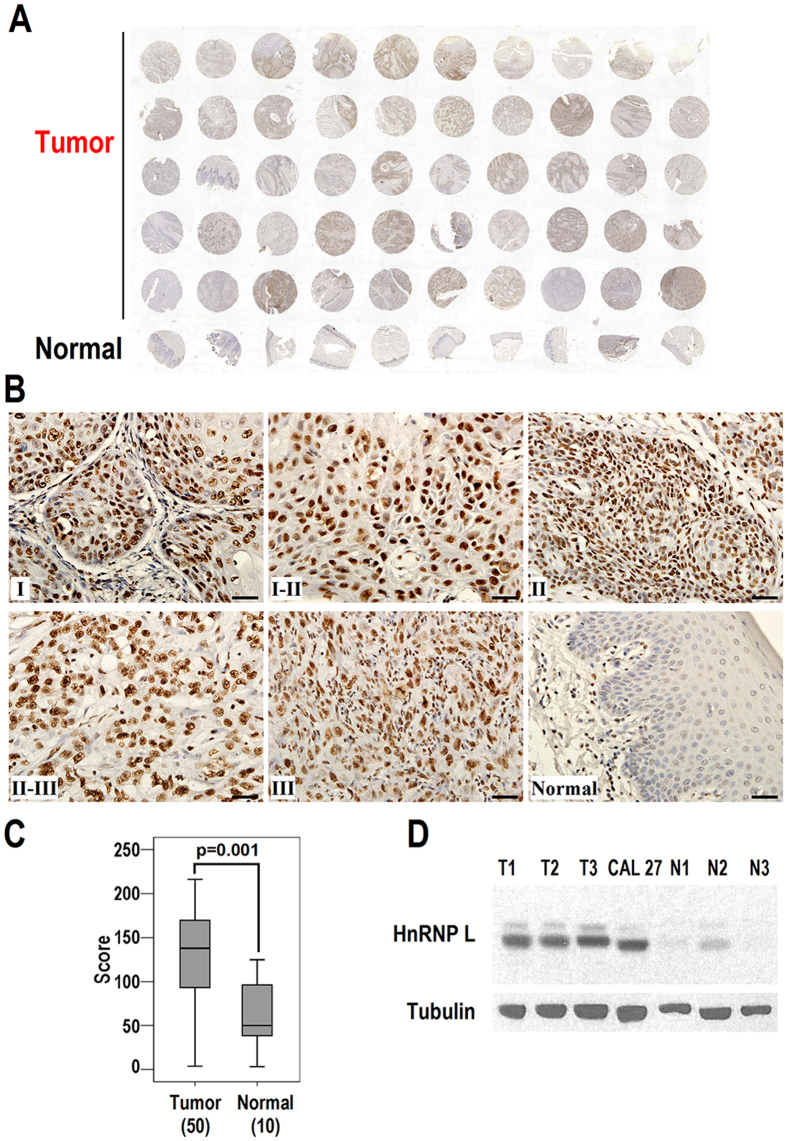
Overexpression of hnRNP L in OSCCs. Immunohistochemical analysis of hnRNP L expression in a commercial OSCC tissue array (including 50 OSCC tumor samples and 10 normal oral mucosa samples). (**A**) Tissue array stained with anti-hnRNP L antibody. The specificity of anti-hnRNP L antibody is confirmed by a negative controls (Figure S1A) and positive control (Figure S1B). (**B**) Representative immunohistochemical staining of hnRNP L in OSCCs with different grades, or normal oral mucosal epithelium. Scale bar is 20 μm. (**C**) Box plot comparing immunostaining scores of hnRNP L between tumor and normal tissues in the tissue array. (**D**) Western blot analysis of the expression of hnRNP L in primary human oral squamous cancer cells, CAL 27 cells, or normal primary oral mucosal epithelial cells. β-tubulin served as loading control.

First, we analyzed the expression of hnRNP L in OSCC tissues in a tissue array (including 50 OSCC tumor samples and 10 normal oral mucosa samples), which allows us to evaluate the expression patterns of hnRNP L under equivalent test conditions. Immunohistochemical staining showed that the levels of hnRNP L in OSCC samples are significantly higher than that in normal tissues (Fig. 1A–C, p = 0.001). However, the statistic power of our result is low because of the relatively small number of samples in tissue array. Thus, we analyzed the expression of hnRNP L in three primary OSCC cells and three normal primary oral mucosal epithelial cells. In consistent with the tissue array results, primary OSCC cells and an OSCC cell line CAL 27 expressed significantly higher levels of hnRNP L than normal cells (Fig. 1D). This result indicates that hnRNP L is overexpressed in OSCC.

In the present study, the immunohistochemical results showed that hnRNP L is mainly expressed in the nuclei of cells, which is consistent with other’s report^19^. It aggregates in some regions of the nucleus and forms speckle-like structures in tumor cells. Expression level of hnRNP L varies significantly in different tumor cells. In normal oral mucosal tissues, weak-to-medium expression levels of hnRNP L were measured. In contrast to epithelium, many strong stained cells were found in mesenchymal tissues of normal or tumor samples, thus indicating that hnRNP L may play some roles in connective tissues (Fig. 1B).

### HnRNP L is involved in OSCC cell growth, cell cycle progression, and apoptosis

**Figure 2:**
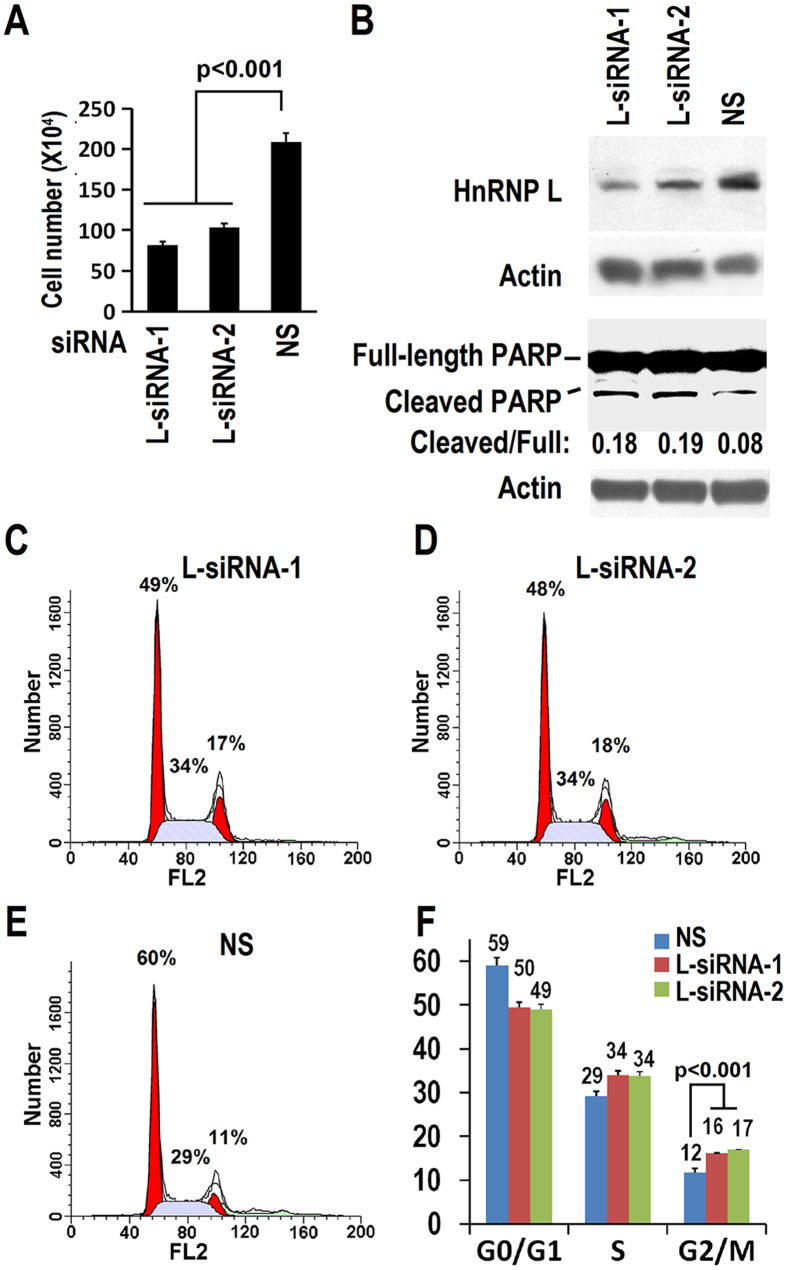
HnRNP L is required for CAL 27 proliferation. (**A**) Knockdown of hnRNP L inhibited CAL 27 cell growth. CAL 27 cells (2 × 10^5^ cells/well) were seeded into 12 well plates and transfected by 20 nM hnRNP L siRNAs or non-specific siRNA (NS) on Day 0. Cells were passed and transfected again on Day 2. Cell numbers were counted on Day 4. Values represent means ± SE. (**B**) Western blot displayed knockdown efficiency of hnRNP L and the cleavage of PARP. The ratio PARP cleaved/full is an index of apoptosis, which was calculated based on the ratio of band intensities of cleaved *vs* full length PARP. β-actin served as loading control. (**C–F**) HnRNP L is involved in cell cycle progression. (**C–E**) Cell cycle analysis of CAL 27 cells treated with L-siRNA-1 (**C**), L-siRNA-2 (**D**), or non-specific (NS) siRNA (**E**). Cells were transfected twice as in (**A**). Results show one representative experiment of four. Data were analyzed by Modfit LT software and drawn as column charts by EXCEL. (**F**) Summary and statistical analysis of four independent experiments. Data are means ± SE.

Next, we analyzed the function of hnRNP L in OSCC cells. We knocked down hnRNP L efficiently with two specific siRNAs targeting different regions of hnRNP L mRNA (Fig. 2B). Both of these two hnRNP L specific siRNAs significantly inhibited the growth of OSCC cell line CAL 27 (Fig. 2A, Figure S4). Cell cycle analysis showed that knockdown of hnRNP L increased the proportion of cells in G2/M phase by 1.33-fold or 1.42-fold (from 12% to 16% or 17%) compared with control, which suggested a moderate G2/M arrest in hnRNP L-reduced cells. (Fig. 2C–F). These results indicate hnRNP L is involved in OSCC cell growth and cell cycle progression.

It has been reported that hnRNP L represses apoptosis^20^. In this study, we also found that knockdown of hnRNP L increased the cleavage of Poly (ADP-ribose) polymerase-1 (PARP) by more than two-fold (Fig. 2B) compared with control, indicating a slightly increased apoptosis induced by hnRNP L knockdown. PARP plays vital roles in the process of apoptosis and is cleaved during apoptosis by caspase-3. Cleaved PARP serves as a marker of cells undergoing apoptosis^21^. This result indicates that hnRNP L is also associated with apoptosis in OSCC cells and regulates cell growth.

### HnRNP L is involved in OSCC cell migration

**Figure 3:**
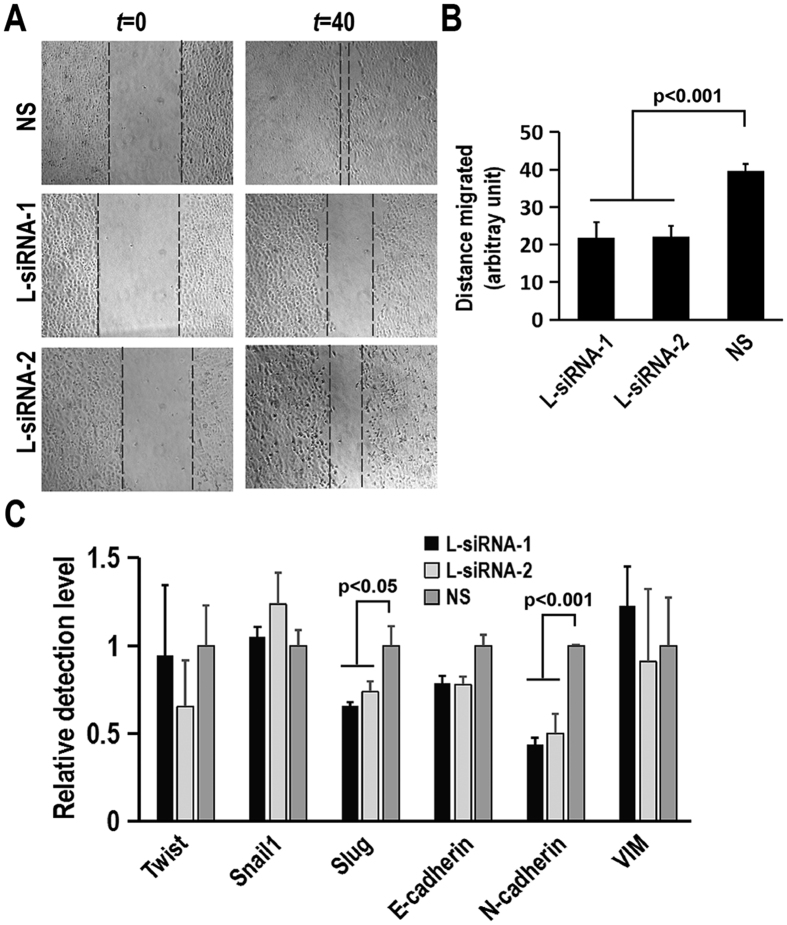
Involvement of hnRNP L in cell migration. CAL 27 cells were treated with siRNA twice and grew to 95-100% confluence. The monolayer of cells were wounded and washed thrice with PBS on 0 h (*t* = 0). Wound healing was photographed at 40 h (*t* = 40). The cell migration was expressed as distance (arbitrary unit) traveled by cells. (**A**) Representative images of CAL 27 cell migration affected by siRNA treatment. (**B**) Summary and statistical analysis of three independent experiments. Data are means ± SE. (**C**) HnRNP L regulates the expression of epithelial-mesenchymal transition (EMT) related-genes. CAL 27 cells were transfected with siRNA twice in an interval of 48 hours. The expression levels of Twist, Snail1, Slug, E-cadherin, N-cadherin and Vimentin (VIM) were analyzed by real-time quantitative RT-PCR.

Invasion and metastasis are two of the hallmarks of cancer^22^. We analyzed the effects of hnRNP L depletion on OSCC cell migration. Wound-healing migration assay showed that cells treated with hnRNP L siRNA migrated significantly slower than control cells (Fig. 3A,B). Western blot assay confirmed that the expression levels of hnRNP L in hnRNP L siRNA treated cells remained significantly lower than those in control cells for two days after serum starvation (Figure S2). Moreover, we analyzed the effect of hnRNP L on the expression of epithelial–mesenchymal transition (EMT)-related genes to understand how hnRNP L regulates cell migration. Results showed that knockdown of hnRNP L significantly reduced the expression levels of Slug and N-cadherin (Fig. 3C). Slug and Snail have been suggested to induce cell movement and EMT^23^. These results indicated that hnRNP L might regulate cell migration *via* controlling the expression of Slug. However, knockdown of hnRNP L had no effect on the expression of E-cadherin (Supplementary Figure S3).

### HnRNP L is required for the tumorigenesis of OSCC cells *in vivo*

**Figure 4:**
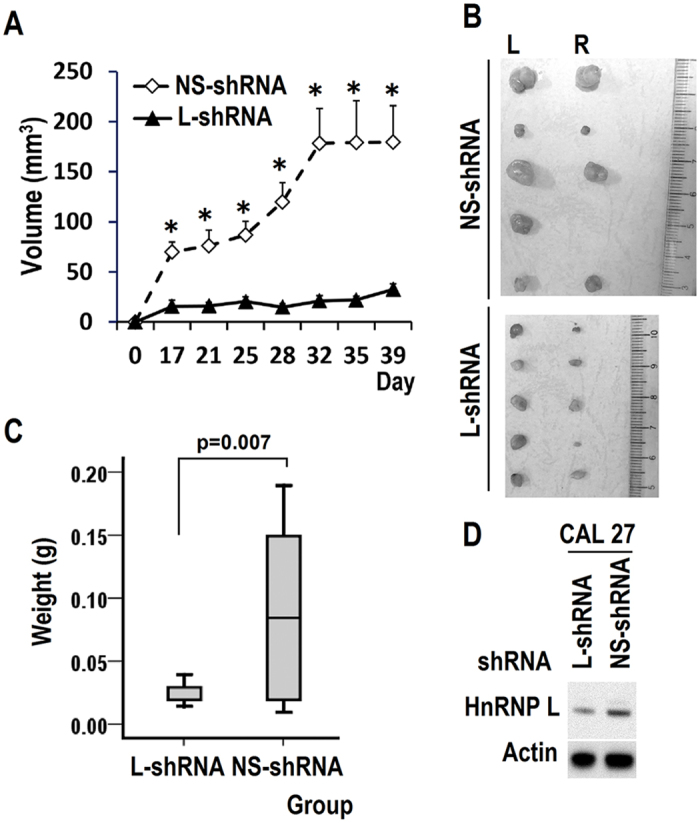
HnRNP L is required for tumorigenesis of OSCC cells *in vivo*. (**A**) 1.5 × 10^6^ CAL 27 cells stably transfected with hnRNP L shRNA (L-shRNA) or non-specific (NS) shRNA were implanted by dorsal subcutaneous inoculation at both sides of nude mice (5 mice per group). Tumor sizes were monitored every 3 to 4 days. Tumor volume was calculated as (length × width^2^) π/6. Data are means ± SE. *p < 0.01. (**B,C**) Tumors were dissected out (L, left; R, right) and weighed on day 39. The boxplot represents the distribution of tumor weight in each group of mice. (**D**) The total protein samples of cells were collected at the time of injection. Western blot displayed knockdown efficiency of hnRNP L. β-actin served as loading control.

We stably transfected CAL 27 cells with hnRNP L or NS shRNA to evaluate the effect of hnRNP L knockdown on the tumorigenesis of OSCC *in vivo*. Results showed that knockdown of hnRNP L significantly retarded tumor formation and growth (Fig. 4A,D). Cells with hnRNP L knockdown showed significantly smaller tumors compared with control cells (Fig. 4B,C). Thus hnRNP L is important for the oncogenic potential of OSCC cells.

### HnRNP L regulates the expression of oncogene SRSF3

**Figure 5:**
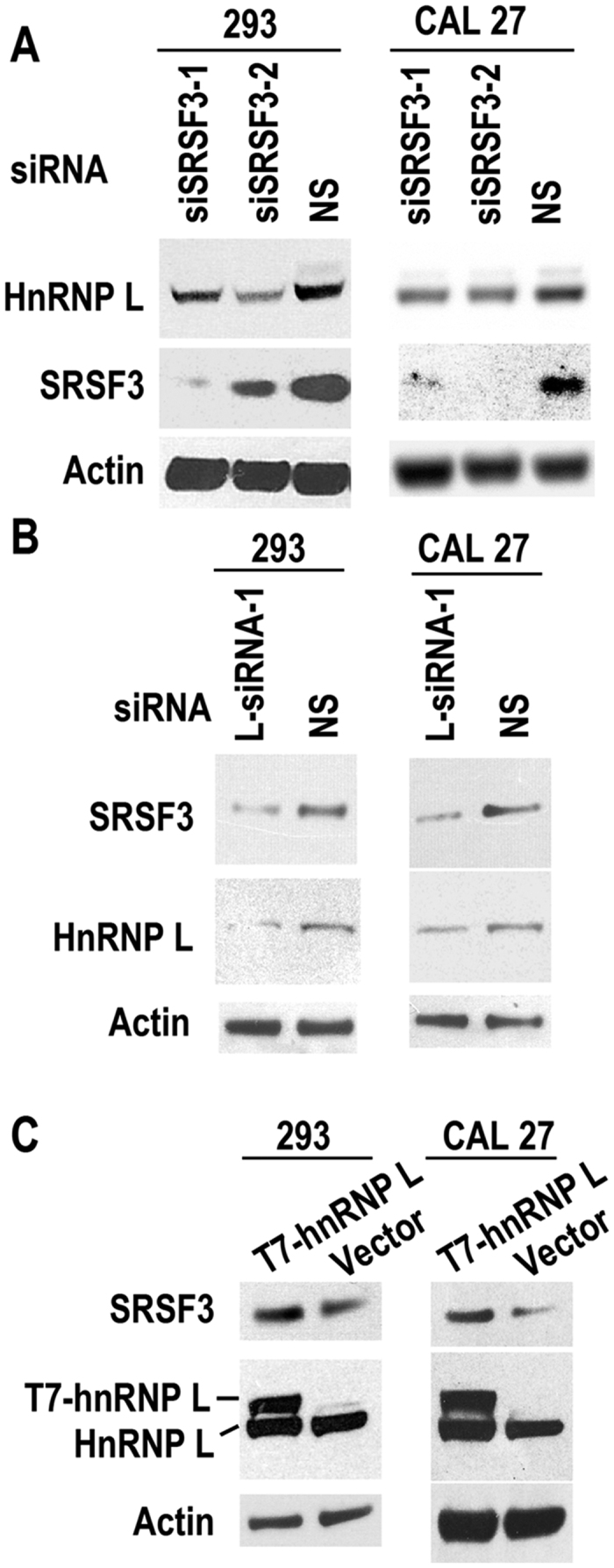
Oncogene SRSF3 is the target of hnRNP L. There is a mutual regulation between SRSF3 and hnRNP L. (**A**) CAL 27 or 293 cells were treated with siSRSF3-1, siSRSF3-2 or none-specific (NS) siRNA. Knockdown of SRSF3 reduced the expression of hnRNP L in both CAL 27 and 293 cells. (**B**) CAL 27 or 293 cells were treated with hnRNP L or none-specific (NS) siRNA. Knockdown of hnRNP L reduced the expression of SRSF3 in both CAL 27 and 293 cells. (**C**) CAL 27 or 293 cells were transfected with T7 tagged hnRNP L or vector plasmid. Overexpression of hnRNP L promotes the expression of SRSF3 in both CAL 27 and 293 cells.

SRSF3 (also called SRp20) is a splicing factor, belonging to SR protein family^24^. It has been proved to be an oncogene^5^ and overexpressed in multiple cancers, including OSCC^25^. We searched the targets of SRSF3 in our previous splice array data (NCBI GEO accession no. GSE22149), in which osteosarcoma U2OS cells were treated with anti-SRSF3 or non-specific (NS) siRNA^26^. In the present study, we found that the expression level of hnRNP L decreased upon SRSF3 knockdown in U2OS cells (fold change: −1.98, p = 0.00516). This result could also be verified in CAL 27 and 293 cells (Fig. 5A) by western blot.

**Figure 6:**
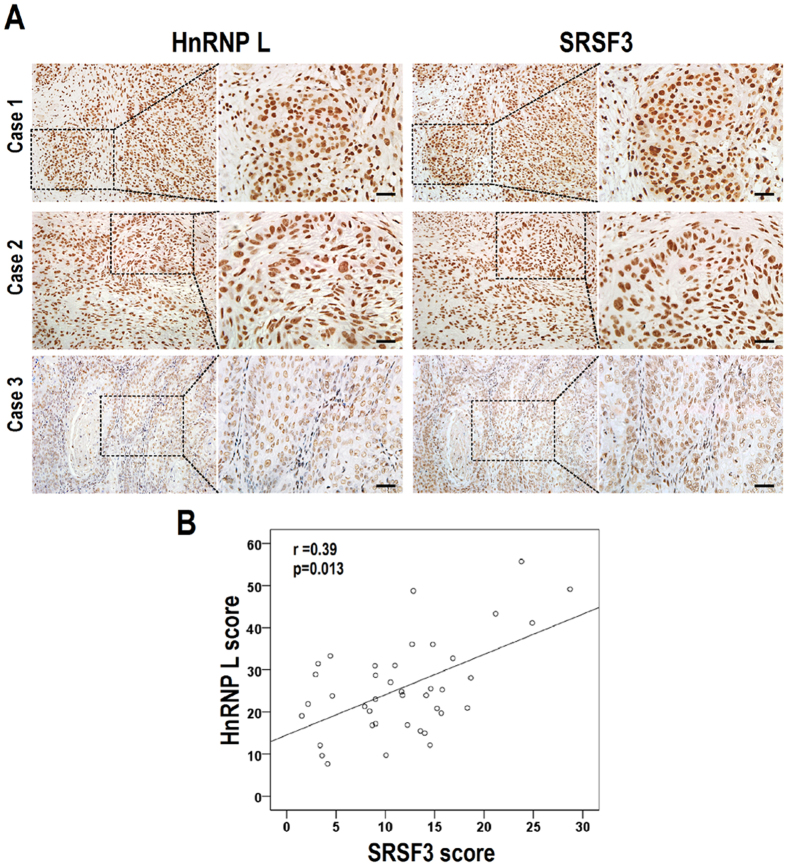
SRSF3 protein expression correlates positively with hnRNP L expression in OSCC tissues. (**A**) Representative immunohistochemical staining of hnRNP L and SRSF3 in the serial sections from the same patients. Case 1 and 2 express high levels of both hnRNP L and SRSF3; case 3 expresses low level of both proteins. Scale bar is 20 μm. (**B**) A significant positive correlation was observed between hnRNP L and SRSF3 expression levels (Spearman’s correlation coefficients: r = 0.39, p = 0.013). Each dot represents a single tumor.

**Figure 7:**
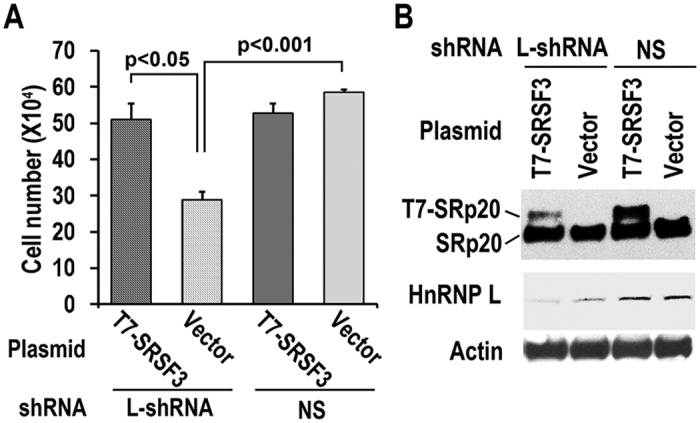
Overexpression of SRSF3 rescued cell growth inhibition induced by hnRNP L knockdown in CAL 27 cells. (**A**) CAL 27 cells stably transfected with hnRNP L shRNA (L-shRNA) or non-specific (NS) shRNA were seeded into 12 well plates (1.5 × 10^5^ cells per well) and transfected by T7-SRSF3 expression plasmid or vector control plasmid. Forty-eight hours later, cell number was counted. Values represent means ± SE. (**B**) Western blot displayed knockdown efficiency of hnRNP L and overexpression of exogenous T7 tagged SRSF3. β-actin served as loading control.

Moreover, we found that knockdown of hnRNP L also reduced the expression of SRSF3 in both CAL 27 and 293 cells (Fig. 5B). Overexpression of T7 tagged hnRNP L promoted the expression of SRSF3 in these two cell lines (Fig. 5C). These results suggested a mutual regulation between hnRNP L and SRSF3. Moreover, the expression levels of both hnRNP L and SRSF3 in OSCC tissues were analyzed. We found that the expression levels of hnRNP L were moderately positively correlated with the expression levels of SRSF3 (r = 0.39, p = 0.013) in OSCC tissues (Fig. 6). In addition, overexpression of T7 tagged SRSF3 in hnRNP L-reduced CAL 27 cells was able to rescue cell growth (Fig. 7). This result indicated that SRSF3 is a target of hnRNP L in cell growth. All these findings suggested that hnRNP L participates in tumorigenesis by regulating the expression of oncogene SRSF3.

### HnRNP L regulates the alternative splicing of SRSF3 exon 4

**Figure 8:**
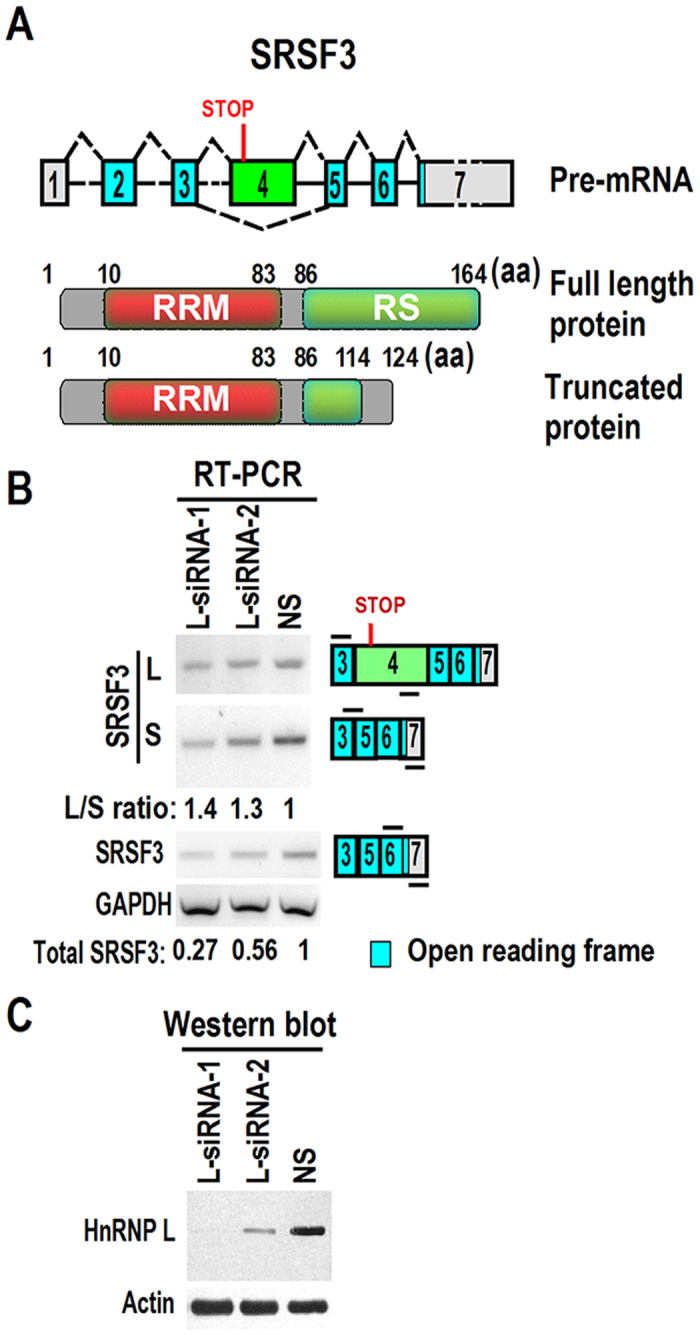
HnRNP L regulates both alternative splicing and transcription of SRSF3 mRNA. (**A**) Schematic diagram of the alternative splicing of exon 4 and functional domains of SRSF3. Inclusion of exon 4, which contains an in-frame stop codon, will cause degradation of SRSF3 transcript or encode a truncated SRSF3. The transcript without exon 4 encodes the full-length SRSF3. Note: aa, amino acids. RRM: RNA recognition motif. RS: arginine/serine-rich domain. (**B**) CAL 27 cells were treated with anti-hnRNP L or none-specific (NS) siRNA twice in a 48-hour interval. Alternative splicing of SRSF3 exon 4 was analyzed by RT-PCR. L/S ratio represents the inclusion levels of exon 4 in mature mRNAs, which was calculated based on the ratio of band intensities of long vs short isoforms. Whole transcriptional level of SRSF3 was analyzed by a pair of primers located at exon 6 and 7, respectively. GAPDH served as the loading control. Diagrams on the right of (**B**) show the structures of SRSF3 pre-mRNA and spliced products. Short lines above or below exons represent primer positions. (**C**) Western blot displayed knockdown efficiency of hnRNP L. β-actin served as loading control.

SRSF3 has an alternative exon 4, which contains an in-frame stop codon. Therefore, inclusion of exon 4 will cause degradation of SRSF3 transcript by nonsense-mediated mRNA decay (NMD) or encode a truncated SRSF3, which is missing the important RS functional domain^27^ (Fig. 8A). SRSF3 contains an RNA recognition motif (RRM) in the N-terminus and an arginine/serine-rich domain (RS) at the C-terminus. RS domain interacts with other proteins and facilitates recruitment of spliceosomal components. Knockdown of hnRNP L in CAL 27 cells also resulted in a decrease at whole transcription level of SRSF3 (measured by primers targeting to exon 6 and 7). However, the relative inclusion levels of exon 4 (L/S ratio in Fig. 8B) increased compared with NS siRNA control. These results indicated that hnRNP L regulates both transcription and alternative splicing of oncogenic splicing factor SRSF3.

## Discussion

OSCC remains one of the most common human cancers with around 500,000 new cases diagnosed each year worldwide^28^,^29^. The mortality of OSCC is still high despite progresses in treatments because the mechanisms of tumorigenesis remain largely unknown. Aberrant alternative splicing of pre-mRNA is increasingly considered as one of the causes of tumorigenesis^30^. Splicing factors are key regulators of alternative splicing of pre-mRNA and have attracted increasing attention in tumorigenesis studies.

Qi *et al*.^18^ found that hnRNP L is up-regulated in esophageal cancer cells compared with untransformed cells in a proteomic analysis. In consistent with their result, we found that OSCC tissues expressed more hnRNP L than normal oral mucosal epithelial tissues in an OSCC tissue array by using immunohistochemistry. Interestingly, many mesenchymal cells in both tumor and normal connective tissues express high levels of hnRNP L. Thus, hnRNP L may have important different functions in soft tissues compared with normal epithelial tissues.

In our previous study, we have demonstrated that the splicing factor SRSF3 is an oncogene and required for G2/M progression of cell cycle^5^. Goehe *et al*. indicated that hnRNP L inhibits apoptosis and promotes the tumorigenic capacity of lung cancer cells^17^. We also found that knockdown of hnRNP L increased the cleavage of PARP. Moreover, our present study further indicated that hnRNP L is important for the growth of OSCC cells, and is involved in G2/M cell cycle progression and tumorigenesis *in vivo*. Thus, both SRSF3 and hnRNP L is involved in tumorigenesis.

Interestingly, in the present study, we demonstrated SRSF3 is a target of hnRNP L in cell growth. Overexpression or down-regulation of hnRNP L increased or decreased, respectively, the protein expression level of SRSF3 (Fig. 5). Overexpression of SRSF3 rescued the growth inhibition induced by the knockdown of hnRNP L (Fig. 7). HnRNP L may regulate the expression of SRSF3 *via* several following mechanisms. Firstly, it has been reported that hnRNP L plays roles in chromatin modification^9^, and stabilizes mRNA of vascular endothelial growth factor^15^. We found that knockdown of hnRNP L decreased the expression level of SRSF3 mRNA (Fig. 8B). It is possible that hnRNP L may be involved in the transcriptional initiation or RNA stability of SRSF3 mRNA. Further experiments are required to test these possibilities. Secondly, at the post-transcriptional level, we found that hnRNP L regulates the alternative splicing of SRSF3 exon 4 (Fig. 8B). Taken together, the data suggested that expression of SRSF3 is regulated by hnRNP L at both transcriptional and post-transcriptional levels.

HnRNP L is a multifunctional splicing factor. Gaudreau *et al*. showed that knockout of hnRNP L repressed the migration of T cells^31^. Yau *et al*. found that knockdown of hnRNP L significantly suppressed cell migration and invasion in Hepatitis B virus- related hepatocellular carcinoma^32^. In this study, we also found that hnRNP L is involved in cell migration of OSCC, and two EMT-related genes, Slug and N-cadherin, decreased in hnRNP L-reduced cells. However, E-cadherin remains unchanged after hnRNP L knockdown treatment (Supplementary Figure S3). Further experiments are required to study the functions of hnRNP L in EMT and metastasis. Currently, we only found that hnRNP L affects the migration of OSCC cells.

In summary, our results demonstrate that hnRNP L plays important roles in the oncogenesis of OSCC, and may be a novel potential therapeutic target of OSCC. Nevertheless, further investigations are necessary.

## Methods

### Cells

HEK-293 cell and OSCC cell line CAL 27 cell were grown in Dulbecco’s modified Eagle medium (DMEM; HyClone, USA) supplemented with 10% fetal bovine serum (FBS) and 1% antibiotic-antimycotic (Gibco, USA). OSCC tumor tissues were obtained from the School of Stomatology in Wuhan University. The tissues were washed with phosphate-buffered saline (PBS) containing 1% antibiotic-antimycotic, and then minced and incubated in collagenase (Worthington Biochemical), followed by centrifugation. The precipitants were resuspended in DMEM supplemented with 10% FBS and 1% antibiotic-antimycotic, and then cultured at 37 °C and 5% CO_2_. Tumor cells were purified by depleting fibroblasts with a short treatment by 0.25% trypsin-EDTA (Invitrogen, USA). N1, N2, and N3 normal gingival epithelial cells were obtained from gingival tissues of healthy donors. The tissues were washed with PBS containing 1% antibiotic-antimycotic and incubated at 4 °C overnight with dispase (2.5 mg/mL; Worthington Biochemical, USA). The separated epithelia were digested in 0.25% trypsin for 5 min, and then minced and pass through a cell strainer followed by centrifugation. The pellets were resuspended in keratinocyte growth medium (KGM, Lonza, Switzerland). All tumor cells were cultured with KGM for two days to analyze the expression of hnRNP L in tumor or normal cells. Informed consent was obtained from all participants, and all experimental protocols were approved by the Ethics Committee at the School of Stomatology in Wuhan University. The methods were conducted in accordance with the approved guidelines.

### Plasmids

The hnRNP L gene was amplified by primer 5′ TCTGCGCCGCCATGTCGC 3′ and 5′ CCTGCTCAGATGGGACTCTTCCTA 3′. A T7 tag was added to the 5′ end of hnRNP L open reading frame. T7-hnRNP L fusion gene was cloned into pLVX-IRES-PURO vector. The T7 tagged SRSF3 (T7-SRSF3) expression plasmid was kindly provided by Dr. Zheng Zhi-Ming (National Cancer Institute, USA).

### Animals

Two groups (5 per group) of 6-week-old female nude mice were purchased from Hunan SJA Laboratory Animal Co., Ltd. (China) and were raised in specific-pathogen-free animal laboratory at School of Stomatology in Wuhan University. The animal experiment protocol was approved by the Ethics Committee at School of Stomatology in Wuhan University. All methods were performed in accordance with the relevant guidelines and regulations.

### Tissue Microarray and immunohistochemistry

A human OSCC tissue microarray, which included 50 OSCC and 10 normal oral mucosal tissues, was obtained from Alenabio Co., Ltd. (China). Immunohistochemical staining was performed using the avidin-biotin peroxidase complex method with a Vectastain ABC kit (Vector Laboratories, USA). Monoclonal mouse anti-hnRNP L (clone 4D11) was obtained from Santa Cruz Biotechnology Inc. (USA). Monoclonal mouse anti-SRSF3 (clone 7B4) antibody was obtained from Thermo Fisher Scientific Inc. (USA). Specific staining intensity was divided into 4 levels (0–3). Cell numbers in each level were counted to calculate the percentage based on total count (0–100)%). Staining scores were calculated by multiplying the intensity score (0–3) by the percent of total.

### Patients and Tissue Samples

Forty patients diagnosed with OSCC were involved in this study (Supplementary Table 1). All histologic diagnoses were performed by the Pathology Department in the School and Hospital of Stomatology, Wuhan University. Informed consent was obtained from all participants. All experimental protocols were approved by the Ethics Committee at the School and Hospital of Stomatology in Wuhan University. SRSF3 expression in the tumor tissues was analyzed in our recent publication^33^. The expression of hnRNP L was further analyzed in this study using immunohistochemistry. Specific staining was quantified by using imageJ software^34^. A mean staining value from three representative regions of each sample was calculated.

### RNAi and transfection

Human hnRNP L siRNA 1 (L-siRNA-1), siRNA 2 (L-siRNA-2) and non-specific (NS) siRNA were synthesized by GenePharma (China). The sequences of L-siRNA-1 and L-siRNA-2 are 5′-GAAUGGAGUUCAGGCGAUG-3′ and 5′-CUACGAUGACCCGCACAAA-3′, respectively^9^. Two SRSF3 siRNAs (siSRSF3-1 and siSRSF3-2) were purchased from Ambion and Santa Cruz Biotechnology, respectively. CAL 27 or 293 cells were seeded into 12 well plates (2 × 10^5^ cells per well) and transfected by 20 nM siRNA in the presence of Lipofectamine 2000 (Invitrogen, USA) on Day 0. NS siRNA was used as control. Cells were passed and transfected again on Day 2. Total protein and RNA were also collected on Day 4. In addition, CAL 27 cell numbers were counted on Day 4. Stable knockdown of hnRNP L in CAL 27 cells was mediated by an hnRNP L shRNA (L-shRNA) expression vector (TG312371C, Origene, USA). CAL 27 cells stably transfected with NS shRNA were used as control. In brief, cells were transfected by hnRNP L shRNA or NS shRNA expression vector. Stable transfected cells were selected by puromycin. The knockdown efficiency of hnRNP L in stably transfected cells was verified by western blot.

For plasmid transfection, cells were seeded into 12 well plates on the day before transfection. Half microgram of plasmid was diluted in 50 μL Opti-MEM reduced serum medium and mixed with 1 μL Lipofectamine 2000 diluted in 50 μL Opti-MEM reduced serum medium. After 20-minute incubation, the DNA/lipo mixture was then added to cells.

### MTS assay

CAL 27 cells were cultured at a density of 1 × 10^4^ cells per well in a 96-well plate. Cells were transfected twice with siRNA in ther presence of Lipofectamine 2000 in a 48-hour interval without passage. After 4 days, cell proliferation was analyzed by adding CellTiter 96^®^ Aqueous One Solution Reagent (Promega, USA) to each well according to the manufacturer’s instruction for 2 hours. The cell viability was determined by measuring the absorbance at 490 nm using a plate-reader (Biotek, USA).

### Western blot

Total protein samples were denatured by boiling for 3 min, and then separated in a 10% SDS-PAGE gel, and transferred to a nitrocellulose membrane. Then, the membrane was blotted with the following antibodies: mouse monoclonal anti-hnRNP L (Santa Cruz Biotechnology), monoclonal mouse anti-SRSF3 (clone 7B4, Thermo Fisher Scientific Inc, USA), mouse monoclonal anti-E-cadherin antibody (Santa Cruz, USA), rabbit monoclonal anti-PARP antibody (Epitomics, USA) or horseradish peroxidase-labeled mouse anti-β-actin antibody (Sigma-Aldrich).

### RT-PCR

Total RNA was purified by using Total RNA Miniprep Kit (AxyPrep, USA). One microgram of total RNA was treated with DNaseI (invitrogen, USA) and reverse-transcribed with Moloney Murine Leukemia Virus Reverse Transcriptase (MMLV, Promega, USA) and random hexamers (Promega, USA). PCR were performed by using Taq DNA polymerase (Takara, Japan) with following primer pairs: 5′ GAAGGTGAAGGTCGGAGTC 3′ and 5′ GAAGATGGTGATGGGATTTC 3′ for GAPDH. oGJH213 5′ CCATAGAGAATTACACCTTTGTGTCACTG 3′ (exon 7) and oGJH761 5′ AGTCCTCCACCTCGTCGCAGATCTC 3′ (exon 3 and 5 junction primer) for exon 4-excluded SRSF3; oGJH759 5′ CATGTGAAACGACACCAGCCAAGC 3′ and oGJH211 5′ CTCCCTCTTGGGGTCGTCGC 3′ for exon 4-included full-length SRSF3; oGJH765 5′ ATCGCTGTCTCGGGAGAGAAATCAC 3′ and oGJH213 for total transcriptional level of SRSF3.

### Real-time quantitative RT-PCR (qRT-PCR)

The realtime quantitative RT-PCR was performed in triplicate with All-in-One™ qPCR Mix (SYBR Green method, GeneCopoeia, China) in a 7900HT Fast Real-Time PCR machine (Applied Biosystems, USA). Primers for qRT-PCR are synthesized according to the publication^35^, except a forward primer (5′ CCACCTACAAAGGCAGAAGAGA 3′) for N-cadherin. The primers for Actin reference is 5′ CCCAGCACAATGAAGATCAA 3′ and 5′ ACATCTGCTGGAAGGTGGAC 3′. The relative levels of gene expression were calculated as ΔCt = Ct(gene) - Ct(reference). The fold-change of gene expression was calculated with the 2^−ΔΔ^Ct method.

### Cell cycle analysis

SiRNA-treated CAL 27 cells were collected and prepared by using a COULTER DNA PREP Reagents Kit (Beckman-Coulter, USA) according to the manufacturer’s instruction. Cell cycle analyses were performed using a BD Biosciences FACSCalibur flow cytometer and Modfit LT software (Verity software house).

### Wound-healing migration assay

CAL 27 cells were transfected by 20 nM L-siRNA-1, L-siRNA-2 or non-specific (NS) siRNA on Day 0. Cells were passed and transfected again on Day 2. (Note: NS siRNA-transfected cells were passed at 1:4, whereas L-siRNA-1- or L-siRNA-2-transfected cells were passed at 1:2 on Day 2.) Cells grew to 95–100% confluence on Day 4. The monolayer of cells were wounded with a 200-μL micropipette tip and washed thrice with PBS on 0 h (*t* = 0). Cells were then cultured in serum-free medium. Wound healing was photographed at 40 h (*t* = 40). The cell migration was expressed as distance migrated by cells.

### Tumor induction in nude mice

CAL 27 cells stably transfected with hnRNP L or NS shRNA were implanted by dorsal subcutaneous inoculation at both sides of nude mice (1.5 × 10^6^ cells per side, 5 mice per group). Tumor sizes were monitored every 3 to 4 days. Mice were sacrificed on Day 39. Tumors were dissected out and weighed.

### Statistical analysis

Nonparametric Mann–Whitney test was used to analyze the difference of medians between tumor and normal groups in OSCC tissue array using SPSS software. All two-group statistical comparisons of means were performed with student’s t test (Excel, Microsoft). The correlation coefficient was determined using Spearman’s nonparametric test.

## Additional Information

**How to cite this article**: Jia, R. *et al*. HnRNP L is important for the expression of oncogene SRSF3 and oncogenic potential of oral squamous cell carcinoma cells. *Sci. Rep.* **6**, 35976; doi: 10.1038/srep35976 (2016).

**Publisher’s note**: Springer Nature remains neutral with regard to jurisdictional claims in published maps and institutional affiliations.

## Supplementary Material

Supplementary Information
