# Supplementary material for: HnRNP L is important for the expression of oncogene SRSF3 and oncogenic potential of oral squamous cell carcinoma cells
Source: Sci Rep. 2016 Nov 3;6:35976. doi: 10.1038/srep35976 (PMC5093901; doi:10.1038/srep35976)
Supplement: Supplementary Information [file srep35976-s1.pdf]

Title: HnRNP L is important for the expression of oncogene SRSF3 and oncogenic potential of oral squamous cell carcinoma cells

Authors: Rong Jia, Si Zhang, Miaomiao Liu, Yan Zhang, Yu Liu, Mingwen Fan, Jihua Guo

Supplementary Figure legends

Figure S1

The specificity of mouse monoclonal anti-hnRNP L antibody (Santa Cruz, clone number 4D11) was confirmed by negative controls and positive control. (**A**) We performed IHC staining in a slide containing two serial sections of OSCC tissue. One section was stained by using primary mouse anti-hnRNP L antibody, whereas another section was stained without the primary anti-hnRNP L antibody as negative control. This mouse anti-hnRNP L antibody showed strong signals in the nuclei. Negative control showed no positive staining. (**B**) We found 293 cells express hnRNP L by western blot, thus we also performed immunocytochemistry in 293 cells as positive control. This mouse anti-hnRNP L antibody showed strong signals in the nuclei. Negative control showed no positive staining.

Figure S2

Time course of the expression of hnRNP L after siRNA transfection. CAL 27 cells were transfected with siRNA twice in a 48-hour interval without passage. After 96 hours, cells were starved with serum-free medium for another two days. Total protein samples were collected on Day 4, Day 5, and Day 6. Western blot showed that the protein levels of hnRNP L in L-siRNA treated cells increased on day 5 and day 6 compared with those on day 4, but was still lower than control cells. Relative expression levels of hnRNP L were determined by the density of hnRNP L normalized to β-actin.

Figure S3

HnRNP L knockdown may have no effect on the expression of E-cadherin. CAL 27 cells were treated with anti-hnRNP siRNA (L-siRNA-1 or L-siRNA-2) or non-specific (NS)

siRNA as in Figure 2. The expression of E-cadherin was analyzed by western blot. L-siRNA-1 treatment showed decrease of E-cadherin. However, L-siRNA-1 treatment did not down-regulate E-cadherin, suggesting the decrease of E-cadherin in L-siRNA-2 treated cells is non-specific. Therefore, hnRNP L may have no effect on the expression of E-cadherin.

Figure S4

Analysis of cell proliferation by MTS assay. CAL 27 cells were cultured at a density of $1x10^4$ cells per well in a 96-well plate. Cells were transfected twice with anti-hnRNP L siRNAs or NS siRNA in a 48-hour interval without passage. After 4 days, cell proliferation was analyzed by CellTiter 96® Aqueous One Solution Reagent (Promega, USA). (**A**) HnRNP L knockdown reduced cell viability. Values represent means ± SE. (**B**) Western blot displayed knockdown efficiency of hnRNP L. β-actin served as loading control.

Supplementary table 1, Clinical characteristics of 40 OSCC patients

Figure S1

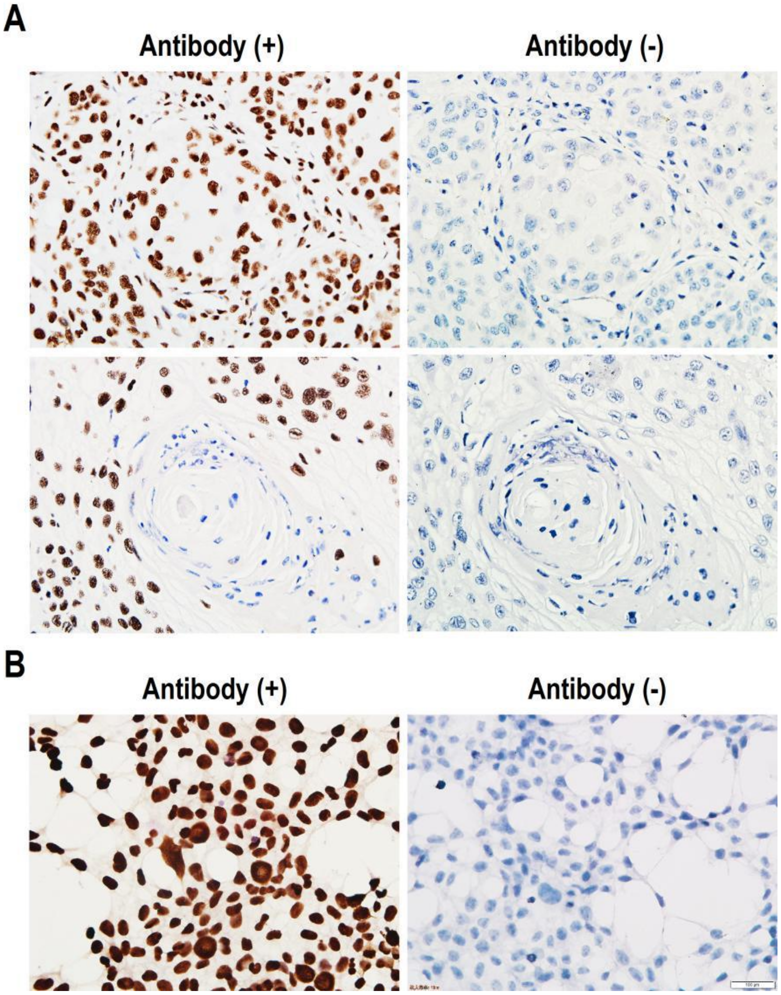

Figure S2

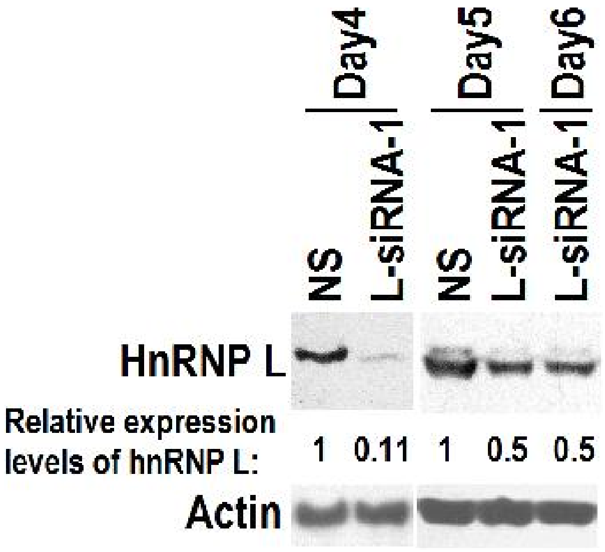
NS
L-siRNA-1
Day4
NS
L-siRNA-1
Day5
L-siRNA-1
Day6
HnRNP L
Relative expression levels of hnRNP L:
1 0.11 1 0.5 0.5
Actin

Figure S3

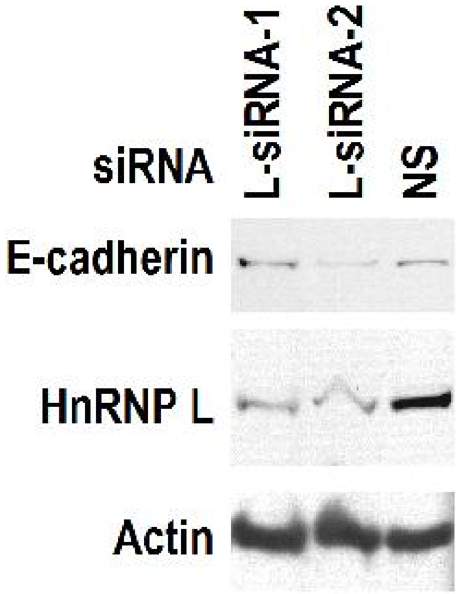

Figure S4

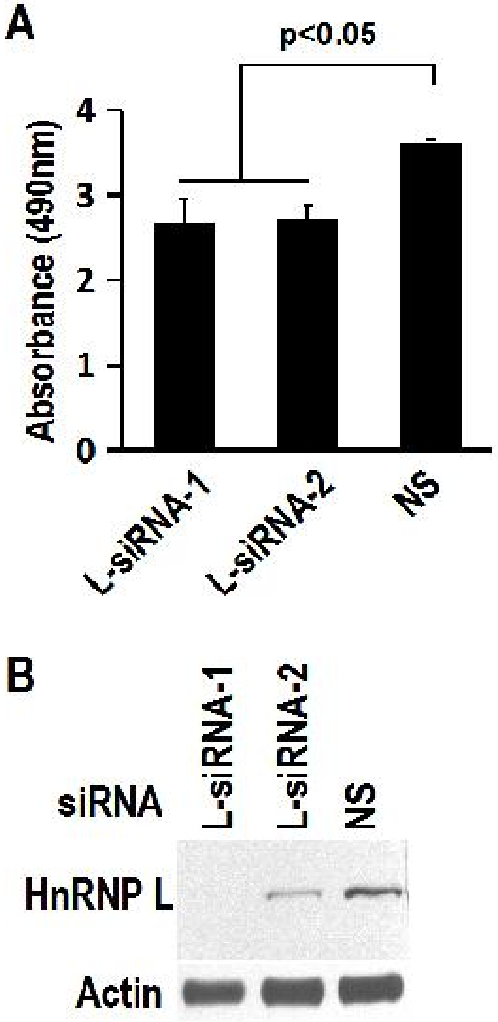
A
p<0.05
Absorbance (490nm)
4
3
2
1
0
L-siRNA-1
L-siRNA-2
NS
B
siRNA
L-siRNA-1
L-siRNA-2
NS
HnRNP L
Actin

Supplementary table 1, Clinical characteristics of 40 OSCC patients

Patients' Characteristics	Number (%)
**Age, years**	
median	54
range	31-79
**Gender**	
male	32(80)
female	8(20)
**Tumor grade**	
I	6(15)
I-II & II	27(67.5)
II-III & III	7(17.5)
**Lymphatic metastasis**	
Positive	12(30)
Negative	28(70)
**Tobacco Smoking**	
Positive	25(62.5)
Negative	15(37.5)
**Drinking**	
Positive	19(47.5)
Negative	21(52.5)
